# *De Novo* Transcriptome Analysis and Detection of Antimicrobial Peptides of the American Cockroach *Periplaneta americana* (Linnaeus)

**DOI:** 10.1371/journal.pone.0155304

**Published:** 2016-05-11

**Authors:** In-Woo Kim, Joon Ha Lee, Sathiyamoorthy Subramaniyam, Eun-Young Yun, Iksoo Kim, Junhyung Park, Jae Sam Hwang

**Affiliations:** 1 Department of Agricultural Biology, National Institute of Agricultural Sciences, Rural Development Administration, Wanju, Republic of Korea; 2 Insilicogen, Inc., Giheung-gu, Yongin-si, Gyeonggi-do, Republic of Korea; 3 College of Agriculture & Life Sciences, Chonnam National University, Gwangju, Republic of Korea; nanyang technological university, SINGAPORE

## Abstract

Cockroaches are surrogate hosts for microbes that cause many human diseases. In spite of their generally destructive nature, cockroaches have recently been found to harbor potentially beneficial and medically useful substances such as drugs and allergens. However, genomic information for the American cockroach (*Periplaneta americana*) is currently unavailable; therefore, transcriptome and gene expression profiling is needed as an important resource to better understand the fundamental biological mechanisms of this species, which would be particularly useful for the selection of novel antimicrobial peptides. Thus, we performed *de novo* transcriptome analysis of *P*. *americana* that were or were not immunized with *Escherichia coli*. Using an Illumina HiSeq sequencer, we generated a total of 9.5 Gb of sequences, which were assembled into 85,984 contigs and functionally annotated using Basic Local Alignment Search Tool (BLAST), Gene Ontology (GO), and Kyoto Encyclopedia of Genes and Genomes (KEGG) database terms. Finally, using an *in silico* antimicrobial peptide prediction method, 86 antimicrobial peptide candidates were predicted from the transcriptome, and 21 of these peptides were experimentally validated for their antimicrobial activity against yeast and gram positive and -negative bacteria by a radial diffusion assay. Notably, 11 peptides showed strong antimicrobial activities against these organisms and displayed little or no cytotoxic effects in the hemolysis and cell viability assay. This work provides prerequisite baseline data for the identification and development of novel antimicrobial peptides, which is expected to provide a better understanding of the phenomenon of innate immunity in similar species.

## Introduction

Cockroaches (order: Dictyoptera; suborder: Blattaria) are among the known primitive winged insects, with an extremely high diversity of ~4,000 species worldwide. Thirty of these species are considered as household insects [1]. The American cockroach *Periplaneta americana* (Linnaeus) is a synanthropic pest that generally inhabits cosmopolitan to urban areas. Cockroaches survive in warm weather with high moisture conditions as well as in unfavorable environments for humans (i.e., sewers and other human-made habitats) [2]. Accordingly, cockroaches physically transmit several human pathogens and allergens from the environment to human habitations [3]. However, the cockroach has also been a beneficial insect for humans, serving as an established model organism for basic research in the fields of neurobiology [4, 5], cardiophysiology [6], blood clotting mechanisms [7], gut microbial diversity [8, 9], and the discovery of allergenic proteins [10].

Innate immunity is the first line of defense of multicellular organisms against invading microbes such as bacteria, fungi, and viruses. Multicellular organisms thus adapt to microbes via their innate immune system through the rapid synthesis and release of various small peptides known as antimicrobial peptides (AMPs) [11].

In insects, AMPs are synthesized from the fat body and various epithelia, which are secreted into the hemolymph. Through the hemolymph, AMPs are directly supplied to the whole body in the context of microbial infection [12]. Moreover, insect autophagy also actively participates along with the innate immunity to evade the microbial infections, and these mechanisms have been extensively studied in the *Drosophila* model. Furthermore, the signal transaction cascade receptors such as pathogen-associated molecular patterns (PAMPs) and pattern recognition receptors (PRRs) are also activated in response to infection [13, 14]. These combinatorial molecular mechanisms serve to completely protect the insect/host from microbial infection. Since the first insect AMPs were isolated from *Hyalophora cecropia* in 1980 [15], 259 insect AMPs have been functionally annotated and classified according to their structural and physiochemical properties [16]. Furthermore, the effects of AMPs on innate immunity, and their corresponding molecular and metabolite/peptide synthesis mechanisms differ according to their degrees of evolutionary conservation [17].

AMPs have been exploited and developed into effective antibiotic and antimicrobial drugs from a diversity of insect species [18]. In particular, Lee et al. [19] suggested that cockroaches are a good source of antimicrobial agents. They further found that the cockroach (*P*. *americana*) brain tissues showed potent broad-spectrum antimicrobial activities, including against antibiotic-resistant bacteria [20]. AMPs are low-molecular-weight and heat-stable proteins, which are typically cationic and often comprise less than 100 amino acid residues. Despite the large number of AMPs that have been identified from different insect species, little information on their potential applications is available. In general, AMPs are predicted through *in silico* approaches based on their derived characteristics, i.e., similarity in physiochemical and structural properties to known AMPs [10, 21]. Several reports have indicated that AMPs can be expressed either constitutively or can be induced upon pathogenic challenge [22]. Alternatively, massive developments in high-throughput sequencing technologies have presented a more efficient method for genomic characterization of a species [23]. However, based on the few studies conducted to date, the genetic resources of cockroaches are scarce [1, 21, 24, 25]. Recently, the transcriptome of the German cockroach (*Blattella germanica*) was reported using next-generation sequencing (NGS) technology, which led to the identification of genes that putatively encode detoxification enzyme systems, insecticide targets, key components in systematic RNA interference, and the immunity and chemoreception pathways [26]. Therefore, identifying new insect AMPs may provide insight into natural interactions between pathogens and proteins. In this present study, we sequenced the *P*. *americana* transcriptome using an NGS platform. Libraries representing control and *Escherichia coli*-immunized *P*. *americana* were systematically analyzed for gene expression profiles along with AMP and allergenic protein prediction. This transcriptome data set and AMPs provide a solid baseline for further functional analysis in *P*. *americana*.

## Materials and Methods

### Animals

This experimental design was approved by the Institutional Animal Care and Use Committee (IACUC) of the National Academy of Agricultural Sciences (approval number: NAAS-1114). Adult American cockroaches were obtained from Kosin University, Busan, South Korea. For immunization, each cockroach was injected by log phase *E*. *coli* (2 × 10^6^ colony forming units [CFU]) suspended in 10 μL of autoclaved 10 mM sodium phosphate buffer (pH 7.4). Cockroaches were reared at 25 ± 1°C for 18 h before total RNA isolation.

### Microorganisms and growth conditions

The bacterial strains *E*. *coli* (KACC 13821, ATCC 11775) and *Staphylococcus aureus* (KACC 10768, ATCC 25923), and the yeast strain *Candida albicans* (KCTC 7121, ATCC 14053) were purchased from the Korean Agricultural Culture Collection (KACC) and Korean Collection for Type Cultures (KCTC). Both bacteria and the yeast were cultivated overnight in tryptic soy broth (TSB; Difco, USA) at 200 rpm in a 37°C shaking incubator to the stationary phase. Bacteria were cultivated for 3 h in fresh TSB medium under the same condition to the log phase. The strains were stored with 15% glycerol at −70°C until use.

### NGS of the cockroach transcriptome

To obtain high-throughput transcriptome data of *P*. *americana*, we implemented Illumina-based NGS sequencing. Total RNA was isolated from *E*. *coli*-immunized (18 h after injection) and non-immunized (Control) adults. Total RNA was quantitated using a Nanodrop spectrophotometer (Thermo Scientific) and its quality was assessed with the RNA 6000 Nano assay kit (Agilent) and Bioanalyser2100 (Agilent). NGS libraries were generated from 1 μg of total RNA using TruSeq RNA Sample Prep Kit (Illumina), according to the manufacturer’s protocol. In brief, the poly-A-containing RNA molecules were purified using poly-T oligo-attached magnetic beads. After purification, the total poly A+ RNA was fragmented into small pieces using divalent cations under elevated temperature. The cleaved mRNA fragments were reverse-transcribed into first-strand cDNA using random primers. Short fragments were purified with a QiaQuick polymerase chain reaction (PCR) extraction kit and resolved with elution buffer for end repair and addition of poly (A). Subsequently, the short fragments were connected with sequencing adapters. Each library was separated by adjoining distinct MID tags. The resulting cDNA libraries were then paired-end sequenced (2 × 101 bp) with the Illumina HiSeq™ 2000 system. The resulting sequences have been deposited at the NCBI Short Read Archive under submission number SRP067419.

### *De novo* assembly and functional annotations

Complete paired-end sequences were obtained as individual FASTQ files (forward and reverse) from the images by using CASAVA v.1.8.2 base-calling software with an ASCII Q-score offset of 64. Adaptor sequences and low-quality bases with PHRED scores (Q) ≤ 20 were removed. Repeat sequences in raw reads were masked by using RepeatMasker (Ver. 4.0.3) against the human and *Drosophila* Repbase database (http://www.girinst.org/). Masked sequences were subjected to *de novo* assembly using CLC Assembly Cell v.4.0 (CLCBio, Inc.; Aarhus, Denmark) with default parameters. Finally, the assembled contigs were subjected to functional annotations with Pendant-Pro^TM^ suite (Biomax, Inc.) under default parameters [27].

### Digital gene expression (DGE) profiling

To characterize the quantitative expression pattern of individual sequences, the clean sequence reads from the two libraries (Control, *E*. *coli*-immunized) were mapped individually to the reference transcriptome by using Bowtie (Ver.0.12.7) with default parameters in TopHat (Ver. 1.3.3). Cufflink (Ver.1.3.0) and Cuffdiff (Ver. 2.0.2) were used to calculate expression profiles with reads per kilobase per million (RPKM) normalization [28]. Genes showing a minimum of 2-fold up- and down-regulation were filtered from the isoforms expression dataset with 1 ≤ log_2_ (fold-change [FC]) values, and the Gene Ontology (GO) annotations were classified using the WEGO webserver [29].

### AMP prediction and classification

The deduced amino acid sequences were subjected to AMP prediction analysis by using a modified bioinformatics strategy. Peptide characteristics of molecular propensity (based on physicochemical properties) and aggregation propensity (*in vitro* and *in vivo)* were determined, and AMP prediction was established using a predefined bioinformatics strategy with parameters defined previously [30]. In addition to this previous strategy, the allergenic propensity of the peptides was also determined using Allerdictor software [31]. Finally, the AMPs were mapped with the CAMP database [32] and classified as novel and known AMPs. To classify the predicted AMPs as novel, sequences were matched to the CAMP database by using two programs: PatMatch (no mismatch) for sequences ≤ 20 bp in length [33] and BLASTP (1E-05) for sequences ≥ 20 bp in length. The BLAST results were filtered with a similarity score ≥ 90. Sequences with observed similarity at the given cutoff values were considered as known AMPs, and others were considered as novel AMPs. Finally, the novel and the known AMPs were manually validated for continuous stretches of amino acids to account for the low-complexity regions and assembly artifacts.

### Peptide synthesis

All putative and novel peptides were selected based on the various prediction tools used previously [28]. The peptides were synthesized using solid-phase peptide synthesis methods at AnyGen Co. Ltd. (Gwangju, Korea). Then, each peptide was purified to >95% by high-performance liquid chromatography, and the purity was confirmed by mass spectrometry analysis. The peptides were dissolved in acidified distilled water (0.01% acetic acid) and stored at −20°C until used in subsequent experiments.

### Antimicrobial activity assay

The radial diffusion assay was performed to test the antimicrobial activities of peptides, as described previously with slight modifications [34]. In brief, bacteria and yeast strains were grown to the mid-logarithmic phase in TSB at 37°C and then washed twice with 10 mM Tris-HCl (containing 5 mM glucose, pH 7.4). A total of 4  × 10^6^ CFU was added to 10 mL of an underlay agarose gel [0.03% (w/v) TSB, 1% (w/v) agarose (Sigma, USA), and 0.02% (v/v) Tween 20 (Sigma, USA) in 10 mM Tris-HCl]. The underlay gel was poured into a 100-mm INTEGRID^TM^ Petri dish. After agarose solidification, 3-mm-diameter wells were punched and 5 μL of each peptide solution was added to each well. Buffer alone was used as a negative control. Plates were incubated at 37°C for 3 h to allow for diffusion of the peptides. The underlay gel was then covered with 10 mL of nutrient-rich agar overlay (6% TSB and 1% agarose in 10 mM Tris-HCl). The antimicrobial activity of a peptide was measured as the diameter of the cleared zone around each well after 12 h of incubation at 37°C. This experiment was repeated at least 3 times and the same results were obtained.

In addition, antimicrobial activities of the peptides were also tested by broth microdilution assays against *E*. *coli*, *S*. *aureus*, and *C*. *albicans*. Briefly, microbes were grown overnight in Mueller-Hinton Broth (MHB) to the onset of the stationary phase with shaking at 200 rpm. The cultures were diluted in fresh MHB to a final concentration of 2 × 10^4^ CFU/mL. A stock solution of each peptide was prepared to a concentration of 640 μg/mL in 0.01% acetic acid, and was then serially diluted two-fold to reach a concentration of 10 μg/mL. After 90-μL aliquots of the microbial suspension were dispensed into each well of a 96-well polypropylene microtiter plate, 10 μL of the peptide solution was added. The antimicrobial activities of the peptides were assessed by measuring the visible turbidity in each well of the plate after 18 h of incubation at 37°C. Minimum inhibitory concentrations (MICs) are expressed as a specific value that caused complete growth inhibition.

### Hemolytic assay

This experiment was approved by the Institutional Animal Care and Use Committee (IACUC) of the National Academy of Agricultural Sciences (approval number: NAAS-1114). The hemolytic activity of the peptides was determined by monitoring the release of hemoglobin from rat erythrocytes at 540 nm. For the hemolytic assay, 20 μL of each peptide solution at a predetermined concentration was added to 180 μL of a 2.5% (v/v) suspension of rat erythrocytes in phosphate-buffered saline (PBS). Melittin (Sigma, USA), a hemolytic and α-helical peptide isolated from bee venom, was used as the positive control. This mixture was incubated for 30 min at 37°C, and 600 μL of PBS was then added to each tube. After 3 min of centrifugation at 10,000 ×*g*, the supernatant was removed, and the absorbance was measured at 540 nm. Evaluations were made from the results of at least three independent experiments, each carried out in triplicate.

### Cell culture and cell viability assay

The human keratinocytes were maintained in Dulbecco’s Modified Eagle Medium supplemented with 10% fetal bovine serum (FBS; Hyclone, USA), penicillin G (100 U/mL), and streptomycin (100 μg/mL) (Invitrogen, USA). Human umbilical vein endothelial cells (HUVECs) were grown on 0.1% gelatin-coated cell culture dishes in M199 medium (Welgene, Korea) supplemented with 20% (v/v) FBS, 3 ng/mL basic fibroblast growth factor (R&D Systems, USA), 5 U/mL of heparin (Sigma, USA), and a penicillin-streptomycin-amphotericin B mixture (100 U/mL potassium penicillin, 100 mg/mL streptomycin sulfate, and 250 ng/mL amphotericin B; Lonza, Belgium) as a complete medium. Cells were cultured at 37°C in a humidified incubator with 5% CO_2_. Cells were plated in 96-well tissue culture plates (2 × 10^4^ cells per well). After 1 day, they were treated with various concentrations (25, 50, 100, and 200 μg/mL) of peptides. Melittin (Sigma, USA) was used as the positive control. After incubation for 24 h, the viability of the cells was assessed by the Cell Titer 96 AQueous One Solution Cell Proliferation Assay according to the manufacturer’s protocol (Promega, USA). The optical density at 490 nm was measured with a microplate reader (Beckman DTX 8800 Multi Detector, USA).

## Results and Discussion

### Sequencing and transcriptome assembly

**Fig 1:**
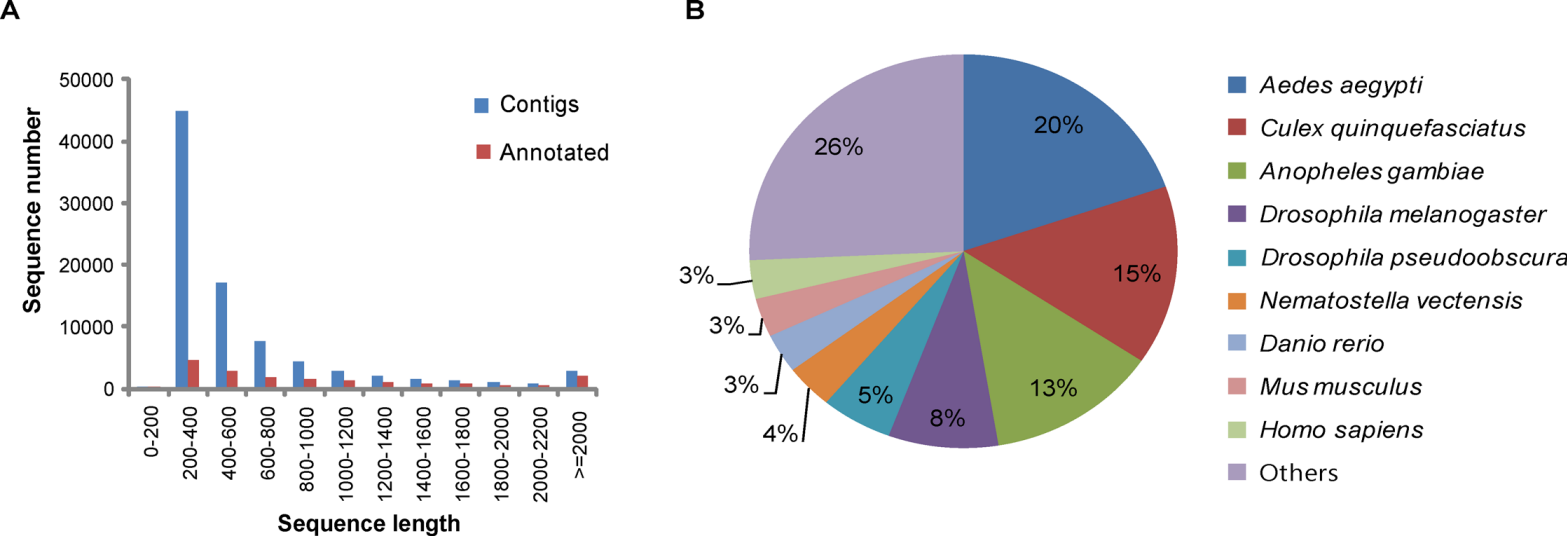
Overview of the *P*. *americana* Annotation. (A) Comparison of sequence length distribution before (blue) and after (red) annotation. (B) Top-hit species distribution of BLAST matches of sequences unique to *P*. *americana*.

The cDNA library prepared from cockroach samples was sequenced using the Illumina HiSeq^TM^ 2000 sequencer. As a result of sequencing, 4,687,932,060 (52,088,134 reads) and 4,781,794,320 (53,131,048 reads) bases were obtained for *E*. *coli*-immunized and non-immunized cockroaches, respectively. We injected live *E*. *coli* into the hemocoel of the cockroaches for immunization, although mixtures of bacteria and fungi should have been employed for the possibility of full induction. A comparative study of differential gene expression is required in future studies to determine the effects of various elicitors. In the present study, we focused on the prediction and experimental validation of novel AMP candidates. None of the novel AMPs was identified among known AMPs that are induced by bacteria and fungi, since we excluded known AMPs after comparison with sequences from the UniProtKB database for the novel AMPs selection procedure. In addition, we employed a naïve sample as a non-immunized control to exclude the expression data of overlapping genes for calculating the maximum fold change of differential gene expression. Initially, the total reads were subjected to preprocessing, as described in the Materials and Methods, resulting in 4,302,302,163 (49,317,908 reads) and 4,380,901,481 (50,270,016 reads) bases, for an average of 91% coverage from raw sequences for the immunized and non-immunized samples, respectively (Table 1). Preprocessed sequences were taken for *de novo* transcriptome assembly by using CLC Assembly Cell v. 4.0. In total, 85,984 contigs were obtained from the assembly, ranging from 200- to 18,078-bp transcripts with an average of 620.8 bp (Fig 1A), which was considered as the draft reference transcriptome for *P*. *americana*.

**Table 1:** Summary of HiSeq and Assembly Statistics from *E*. *coli-*immunized and Non-immunized *P*. *americana*.

Description	Samples	Reads	%	Bases	%	Avg. Length (bp)
Raw sequences	1.TG1110R2526_l1(*E*. *coli*-immunized)	52,088,134	100.0	4,687,932,060	100	90.0
	2.TG1110R2527_l1(non-immunized)	53,131,048	100.0	4,781,794,320	100	90.0
Pre-Processed sequences	1.TG1110R2526_l1(*E*. *coli*-immunized)	49,317,908	94.6	4,302,302,163	91.77	82.5
	2.TG1110R2527_l1(non-immunized)	50,270,016	94.6	4,380,901,481	91.62	82.5
*De novo* assembly	Contig	85,984		53,382,468		620.8
Repeat mask	Contigs_Masked	85,608		44,222,145		516.6

### Functional annotation of unigenes

**Fig 2:**
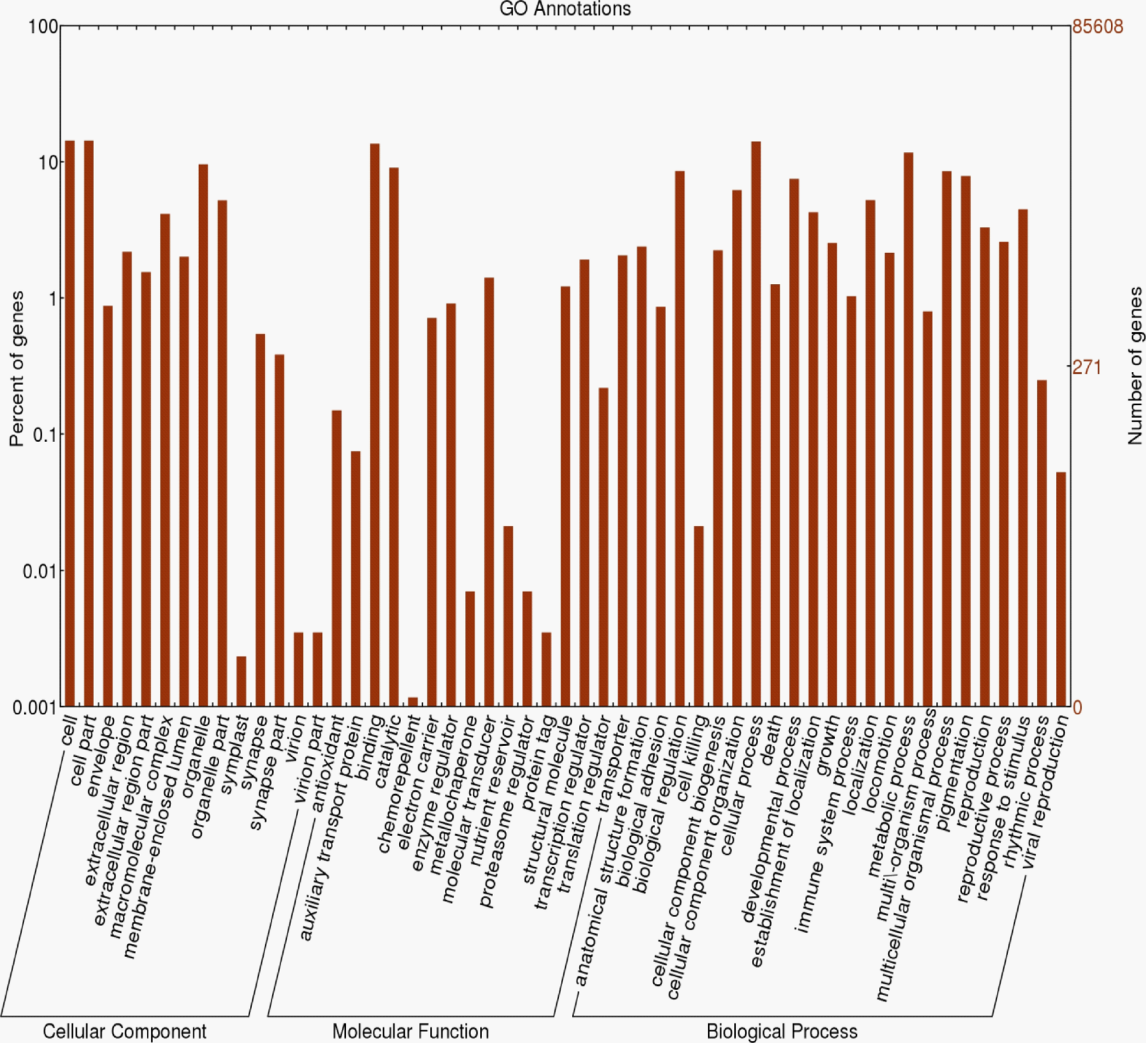
Gene Ontology (GO) Classification of the *P*. *americana* Transcriptome. The histogram of the GO annotation was generated automatically using the web histogram tool WEGO (http://wego.genomics.org.cn/cgi-bin/wego/index.pl) based on the most recent GO archive available. The results are summarized into three main GO categories: cellular component, molecular function, and biological process. The right y-axis indicates the number of genes in a category. The left y-axis indicates the percentage of a specific category of genes in that main category. One gene could be annotated into more than one GO term.

The standardized automated software suite Pendant-Pro (Biomax Informatics) was used to annotate the transcripts. Initially, the assembled transcripts were subjected to repeat masking with a human repeat library, resulting in 44,222,145 reads (85,608 contigs), and masked sequences were subjected to Pendant-Pro with default parameters to obtain the annotations. In total, 17,744 (20.7%) sequences were annotated from 13,726 UniProt protein sequences (Table 2) and the remaining sequences were unannotated (most sequences < 300 bp were not annotated well) (Fig 1A). More than 60% of the annotated sequences were homologous to proteins from mosquitoes (*Aedes aegypti*, *Anopheles gambiae*, *Culex quinquefasciatus*), flies (*Drosophila melanogaster*, *Drosophila pseudoobscura*), and mammals (human and mouse) (Fig 1B). Further, the annotated transcripts were grouped into GO subcategories, i.e., biological process (BP), molecular functions (MF), and cellular components (CC), from level-2 GOs. The GO terms cell; cell part; organelle (in CC); binding, catalytic, and transcription regulators (in MF); and cellular process, metabolic process, and pigmentation (in BP) were shown to be the top 3 clusters (Fig 2).

**Table 2:** Summary Statistics of Gene Ontology Categories.

Groups	Category	Contigs (%)	
A. BLAST	Total contigs	85,608 (100)	
	Has UniProt ID	17,744 (20.7)	
	Unique UniProt ID	13,726 (16.0)	
	Category	# Up (%)	# Down (%)
B. Digital Gene Expression	Fold change ≥ 2	848 (1.0)	1,228 (1.4)
	Known UniProt ID	232 (0.3)	448 (0.5)
	Unique UniProt ID	218 (0.3)	373 (0.4)
	Category	# Term	# Contigs (%)
C. Gene Ontology	Biological Process	4,953	11,395 (13.3)
	Molecular Function	2,712	11,822 (13.8)
	Cellular Component	947	10,958 (12.8)

### DGE profile

To analyze the gene expression profiles of *P*. *americana* from the transcriptome data, DGE analysis was performed, as described in the Materials and Methods. In total, 2,076 (2.4%) transcripts were found to be significantly up- and down-regulated with a ≥2-fold change. Among these transcripts, 848 (1.0%) were up-regulated and 1,228 (1.4%) were down-regulated in the immunized condition, which were plotted in a histogram based on the GO categories (S1 Fig).

### *In silico* analysis of allergens and AMPs from *P*. *americana*

Isolation of AMPs from insects has been one of most effective and promising strategies in the development of antimicrobial drugs [18]. For the most part, AMPs have been predicted through computational rather than experimental methods. The primary goal of this study was to predict the AMPs from the transcripts of *P*. *americana* and validate these predictions experimentally. In total, 86 AMPs were predicted to be novel AMPs (Table 3 and S1 Table), 72 were identified as putative AMPs (S2 Table), as defined in the Materials and Methods, and 180 proteins were predicted as allergens (S3 Table). Both the novel and putative AMPs were identified as non-allergenic peptides and are listed in Table 3. Among the putative AMPs, 54 are known to function as antibacterials, 5 are antifungals, and 3 are antivirals (S1 Table). Three of these transcripts were annotated as being related to the immune response (ISGCock_Contig01_0792, ISGCock_Contig04_0023, and ISGCock_Contig08_4679), 20 transcripts were annotated as being involved in protein binding, and none of the novel AMPs was annotated. The allergenic proteins were grouped into GO subcategories (S2 Fig). Previously known allergen proteins were only predicted from 233 UniProt database sequences of *P*. *americana*, and 9 were validated [10]. In our predictions, 57 novel transcripts were predicted as allergens. These novel candidates should be useful for progress in anti-allergen development.

**Table 3:** *In Silico* Functional Characterization of Selected Antimicrobial Peptides (AMPs).

Program	Parameter		Filtered
PEPSTATS	Length	≤ 50mer	18,309
	Charge	>0(+)	64,172
	pI	8 ≤ pI ≤ 12	51,872
AMPA	Stretch No.	≥1	45,751
Known AMP	BLASTP (E-value)	1.00E^-05^	83,350 (42)
	PATMATCH	No mismatch	30
CAMP	AMP (Discriminant analysis)	TRUE (score < -0.251)	30,534
	AMP (SVM)	TRUE (no score)	26,948
TANGO	AGG	AGG ≤ 500	37,092
	HELIX	0 ≤ HELIX ≤ 25	38,526
	BETA	25 ≤ BETA ≤ 100	3,711
AGGRESCAN	Na4vSS	-40 ≤ Na4vSS ≤ 60	81,166
EPESTFIND	Protein cleavage site	FALSE	40,791
Expression change	NC or Increased	-1 < log_2_ (FC)	64,725
Final filtered AMPs			86

pI, isoelectric point; SVM, support vector machine; NC, no change; FC, fold change.

### Experimental validation of putative and novel AMPs

**Fig 3:**
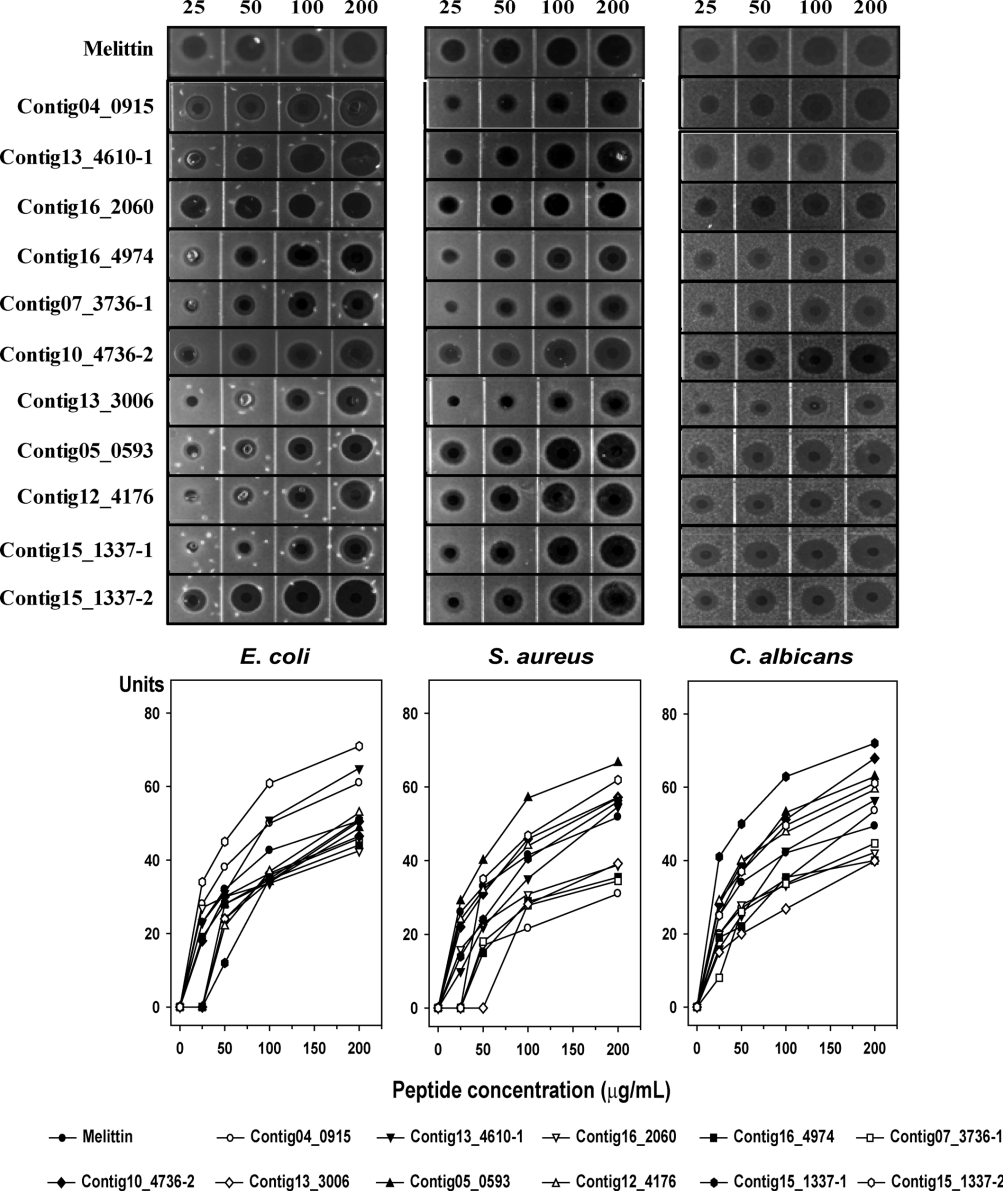
Radial Diffusion Assay. Antimicrobial activities of 11 selected peptides against *E*. *coli*, *S*. *aureus*, and *C*. *albicans* determined by a radial diffusion assay. Peptide concentration (x-axis) was plotted against the diameter of the microbial growth inhibition zone (y-axis) after incubation for 12 h, and is expressed in units (1 mm = 10 units). Melittin was used as a positive control. Mean values were obtained from tests repeated three times.

Experimental validation is required to examine the accuracy of any putative and novel AMPs identified. The 86 peptides were sorted according to fold-change in expression, and 21 AMPs with potentially high activity were ultimately selected (Table 4). Twenty-five peptides were synthesized according to results of the AMPA server (http://tcoffee.crg.cat/apps/ampa). We chose the active regions based on AMPA stretch due to efficiency and cost of peptide synthesis for development of antimicrobial agents. We tested their antimicrobial activities against Gram-negative bacteria, Gram-positive bacteria, and yeast using a radial diffusion assay (Fig 3). We found antimicrobial activity in 11 synthetic peptides (ISGCock_Contig04_0915, ISGCock_Contig13_4610–1, ISGCock_Contig16_2060, ISGCock_Contig16_4974, ISGCock_Contig07_3736–1, ISGCock_Contig10_4736–2, ISGCock_Contig13_3006, ISGCock_Contig05_0593, ISGCock_Contig12_4176, ISGCock_Contig15_1337–1, and ISGCock_Contig15_1337–2), which increased in a dose-dependent manner. Remarkably, the peptides ISGCock_Contig04_0915, ISGCock_Contig13_4610–1, and ISGCock_Contig15_1337–2 showed stronger antimicrobial activity than melittin in *E*. *coli*. The antimicrobial activities of ISGCock_Contig13_4610–1, ISGCock_Contig10_4736–2, ISGCock_Contig05_0593, ISGCock_Contig12_4176, ISGCock_Contig15_1337–1, and ISGCock_Contig15_1337–2 were greater than that of melittin in *S*. *aureus*. Correspondingly, ISGCock_Contig04_0915, ISGCock_Contig13_4610–1, ISGCock_Contig10_4736–2, ISGCock_Contig05_0593, ISGCock_Contig12_4176, ISGCock_Contig15_1337–1, and ISGCock_Contig15_1337–2 showed strong antimicrobial activity against *C*. *albicans*.

**Table 4:** List of Predicted Antimicrobial Peptides (AMPs) from American Cockroach Transcripts.

Sequence ID	Length	Discriminant	log2(FC)	Sequence	SVM-score	Prediction
ISGCock_Contig04_0915	50	AMP	3.07	ALQICTRNMIDDRLPYVADNVRPGTFIKQQRKQKQQRHHTSGTRKRMAKG	-1.01	non-allergen
ISGCock_Contig02_3734	44	AMP	2.96	KLHEFKLGYPLATNYACAIARDLILHKIYIIHFLHRLRKKLSHY	-1.04	non-allergen
ISGCock_Contig14_1231	41	AMP	2.57	ISYFLFLDFRDIFHSQRRKVNFNAGIHSHKNNNKYKLSSCQ	-1.02	non-allergen
ISGCock_Contig13_3331	47	AMP	2.55	IRFGKFKNLRQKQENRCGDIFKQRQGLETCRHRLQFKIDLYISTNDK	-1.00	non-allergen
ISGCock_Contig13_4610–1	44	AMP	2.55	HLYPCKLNLKLGKVPFHFLNLNHKGKSIMVNQQTCLYYIICQTR	-0.97	non-allergen
ISGCock_Contig13_4610–2	44	AMP	2.55	HLYPCKLNLKLGKVPFHFLNLNHKGKSIMVNQQTCLYYIICQTR	-0.97	non-allergen
ISGCock_Contig01_3774	31	AMP	2.31	PPHMQSPLCAPCKIQGRSIVFRTSIVLVNLN	-1.00	non-allergen
ISGCock_Contig12_2253	23	AMP	2.31	KQRKEGECGQFLTKVNSGKIITG	-1.00	non-allergen
ISGCock_Contig13_4305	36	AMP	2.29	YAHLSNIPIFQVCVCSKVYYIHKHFTNYLRVSKQNC	-1.02	non-allergen
ISGCock_Contig16_2060	34	AMP	2.29	ISHNHLTAASITHVKNRGKYIYMHLKFRKTNVLI	-1.04	non-allergen
ISGCock_Contig13_2121	31	AMP	2.27	LSPHSSNVKRKEHLLSNCKFNFYRLKLIQIP	-0.99	non-allergen
ISGCock_Contig11_1401	41	AMP	2.01	RTSKNYLIITQLKGENLESPKDIRKIIFSNGDRLDCRKSKP	-0.96	non-allergen
ISGCock_Contig16_4974	44	AMP	1.99	RKKVWFIFHVCPKLKQRILSDTHAKNKCRLSPLLIKSTKIKNET	-1.00	non-allergen
ISGCock_Contig07_3736–1	31	AMP	1.88	CNYISFFRKCKNSQSTMYGCHRMNKCVFSSY	-1.02	non-allergen
ISGCock_Contig07_3736–2	31	AMP	1.88	CNYISFFRKCKNSQSTMYGCHRMNKCVFSSY	-1.02	non-allergen
ISGCock_Contig14_0122	26	AMP	1.70	SINRFLQHYNISLYTPYNFIIKKTNF	-1.01	non-allergen
ISGCock_Contig05_0163	35	AMP	1.66	KSILYLLCRDFRDLHKYAALRIVQSPRRTLYNKLN	-1.00	non-allergen
ISGCock_Contig10_4736–1	37	AMP	1.57	LMLCKGFLRHSYKSIHERGTKRGKLCRISRLALSSLP	-0.77	non-allergen
ISGCock_Contig10_4736–2	37	AMP	1.57	LMLCKGFLRHSYKSIHERGTKRGKLCRISRLALSSLP	-0.77	non-allergen
ISGCock_Contig07_2123	19	AMP	1.55	NIYHFFNINKTQFLLITHN	-1.01	non-allergen
ISGCock_Contig13_3006	47	AMP	1.49	ANLLRHKVYGYCVLGPKGSSLGGIHGTWHDHHCSLIQRNPSTSTKGN	-1.00	non-allergen
ISGCock_Contig05_0593	18	AMP	1.48	MKTFLRLYRSLINKVLHV	-1.01	non-allergen
ISGCock_Contig12_4176	30	AMP	1.38	VVGRKHSILNCIPYLKKKKIMRLVESESIG	-1.06	non-allergen
ISGCock_Contig15_1337–1	46	AMP	1.31	KRMKLNAKKLSFCDHLNSYLNLSPTLFIHNSSKQWSHWLWHNGIRI	-1.00	non-allergen
ISGCock_Contig15_1337–2	46	AMP	1.31	KRMKLNAKKLSFCDHLNSYLNLSPTLFIHNSSKQWSHWLWHNGIRI	-1.00	non-allergen

The underlined peptide sequences were synthesized according to results of the AMPA stretches.

Thus, different effects were observed for different strains. ISGCock_Contig15_1337–2 showed the highest antimicrobial activity of the tested peptides in *E*. *coli*, ISGCock_Contig05_0593 showed the highest antibacterial effect in *S*. *aureus*, and Contig15_1337–1 showed the highest antifungal activity of the tested peptides with *C*. *albicans*. Although the antimicrobial activity of AMPs is generally related to the cell membrane components of the microbes known as PAMPs [35], these AMPs showed a broad range (200 μg/mL) of activity toward Gram-negative bacteria, Gram-positive bacteria, and yeast.

We performed additional antimicrobial testing to determine MIC values against *E*. *coli*, *S*. *aureus*, and *C*. *albicans*. Table 5 shows the antimicrobial activities of the selected peptides including melittin as a control peptide. The MICs of melittin for microbes were measured to be between 4 μg/mL and 8 μg/mL. The ISGCock_Contig16_2060, Contig16_4974, ISGCock_Contig10_4736–2, ISGCock_Contig05_0593, and ISGCock_Contig12_4176 peptides showed potent antibacterial activities in *E*. *coli*. Most of the peptides were relatively less potent against *S*. *aureus* except for ISGCock_Contig16_2060, ISGCock_Contig16_4974, and ISGCock_Contig05_0593 compared to their *E*. *coli*-cidal activities. The ISGCock_Contig16_2060, Contig16_4974, ISGCock_Contig05_0593, ISGCock_Contig12_4176, and ISGCock_Contig15_1337–1 peptides showed potent anti-*Candida* activities and the MIC values were equal to the anti-*E*. *coli* activities except for ISGCock_Contig15_1337–1. In contrast, the ISGCock_Contig04_0915, ISGCock_Contig13_4610–1, ISGCock_Contig07_3736–1, ISGCock_Contig13_3006, ISGCock_Contig15_1337–1, and ISGCock_Contig15_1337–2 peptides exhibited higher MIC for most strains, indicating that these peptides may be influenced by the MHB components. Further study is required to elucidate the mechanism and source of the observed antimicrobial activity. Overall, the ISGCock_Contig16_2060, Contig16_4974, ISGCock_Contig05_0593, and ISGCock_Contig12_4176 peptides were prime candidates for development of antimicrobial agents.

**Table 5:** Minimum Inhibitory Concentration (MIC, μg/mL) for Antimicrobial Activity of the Selected Peptides and Melittin.

	*E*. *coli* (ATCC 11775)	*S*. *aureus* (ATCC 25923)	*C*. *albicans* (ATCC 14053)
Melittin	8	4	8
ISGCock_Contig04_0915	64	>64	>64
ISGCock_Contig13_4610–1	64	>64	>64
ISGCock_Contig16_2060	16	16	16
ISGCock_Contig16_4974	16	16	16
ISGCock_Contig07_3736–1	>64	>64	>64
ISGCock_Contig10_4736–2	16	>64	32
ISGCock_Contig13_3006	64	>64	64
ISGCock_Contig05_0593	4	16	4
ISGCock_Contig12_4176	8	>64	8
ISGCock_Contig15_1337–1	>64	>64	16
ISGCock_Contig15_1337–2	>64	>64	64

**Fig 4:**
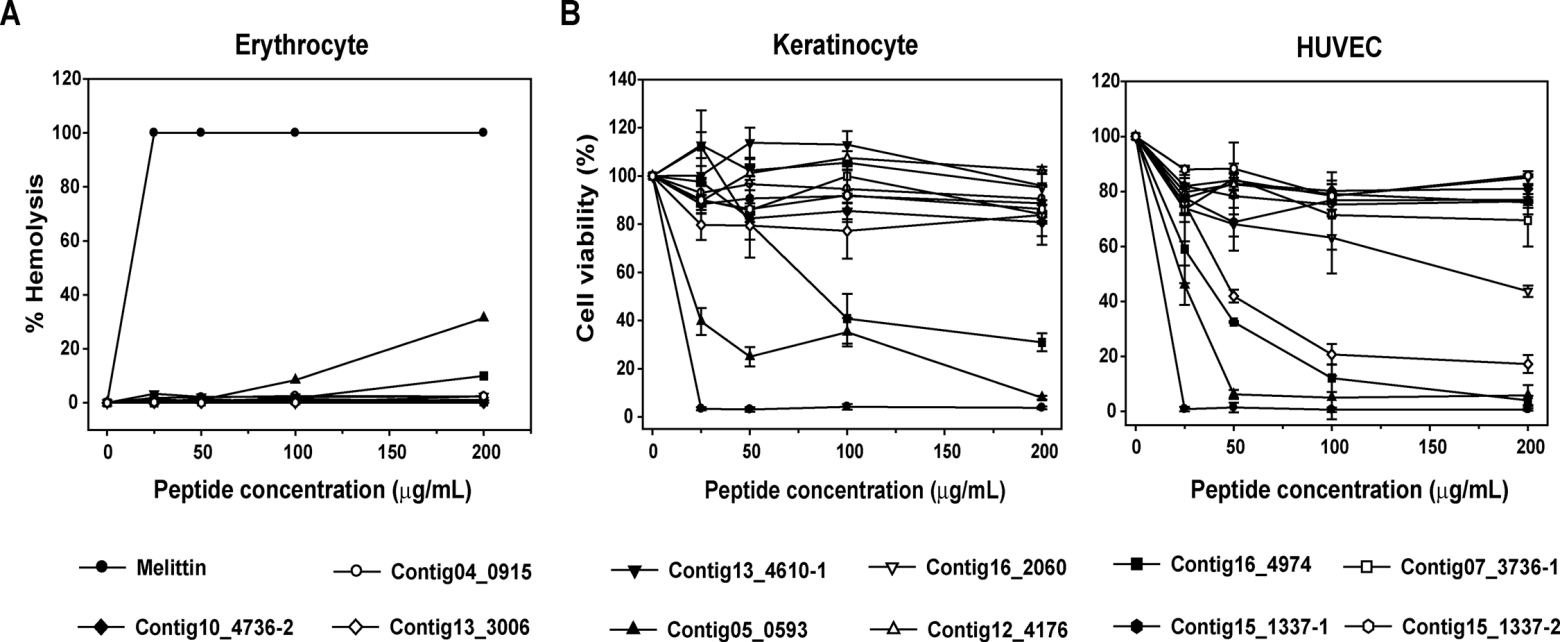
Cytotoxic Effects of 11 Selected Peptides. (A) Hemolytic activity of the peptides. Peptide concentration (x-axis) is plotted against the percentage of hemolysis (y-axis) of rat red blood cells after incubation for 30 min. Melittin was used as the positive control. The percent hemolysis was calculated with the following equation: hemolysis (%) = (A_540_ of sample − A_540_ of peptide-free control)/(A_540_ of 100% control − A_540_ of peptide-free control) × 100. (B) Cell viability of human keratinocytes and human umbilical vein endothelial cells (HUVECs) after peptides treatment. Cell viability was measured by an MTS assay after a 24-h incubation with the indicated amounts of each peptide. Each symbol represents the mean value estimated from triplicate experiments.

The hemolytic effects of the 11 selected synthetic peptides showing antimicrobial activity in the radial diffusion assay are shown in Fig 4A. Melittin lysed 99% of rat red blood cells at a concentration of 25 μg/mL, whereas no hemolytic activity was observed for the 11 synthetic peptides at this concentration and up to 50 μg/mL, although the ISGCock_Contig05_0593 and Contig16_4974 peptides showed relatively strong hemolytic activity even at a high concentration (200 μg/mL). Nevertheless, the hemolytic activities of the peptides ISGCock_Contig13_4610–1, ISGCock_Contig12_4176, ISGCock_Contig15_1337–1, and ISGCock_Contig15_1337–2 were relatively low compared to that of melittin at a concentration of 100 μg/mL. Therefore, the ISGCock_Contig05_0593 and Contig16 _4974 peptides are thought to be effective at doses less than 100 μg/mL, indicating their potential as therapeutic agents against a vast array of microbial infections (Fig 4A). In addition, we investigated the cell viabilities of normal human cell lines (keratinocytes and HUVECs) after treatment with the selected peptides for 24 h at the same concentration of the hemolysis assay. Most of the peptides did not decrease cell viabilities of the cell lines except for the ISGCock_Contig16_4974 and Contig05_0593 peptides together with the ISGCock_Contig16_2060 and Contig13_3006 peptides in HUVECs (Fig 4B). These two peptides (ISGCock_Contig16_4974 and Contig05_0593) showed hemolytic activity in the hemolysis assay and the data are consistent with the MTS assay results, except for the ISGCock_Contig16_2060 and Contig13_3006 peptides, which suggests that these peptides have a specific cytotoxic effect on eukaryotic cells. In contrast, normal human cells were more susceptible to melittin treatment even at the lowest concentration. Melittin has strong and broad antimicrobial spectrum, but the peptide lacks selectivity in normal cells. The purpose of this experimental study is to find novel peptides, which have potent antimicrobial activities with little or no cytotoxicity. Thus, these data indicate that the selected peptides are useful for the development of novel antimicrobial agents.

## Conclusions

Microbial resistance towards antibiotics threatens the effective prevention and treatment of a wide range of infections caused by bacteria, parasites, viruses, and fungi. In recent years, intensive studies have been undertaken towards the development of more effective antimicrobial drugs. AMPs are vital components of innate immunity that can rapidly respond to diverse microbial pathogens. Insects, as a rich source of AMPs, have attracted considerable research attention with respect to both understanding the insect’s immune system and searching for new molecular models for anti-infective drug design [1, 6, 11].

Here, we have shown the effectiveness of a combination of *in silico* and *in vitro* approaches to identify the putative and novel AMPs in *P*. *americana*. We performed *de novo* transcriptome sequencing of *E*. *coli*-immunized and non-immunized *P*. *americana* and selected 86 AMPs by combining the transcriptome with the successive assembly strategies. We further validated the antimicrobial and hemolytic effects of 11 selected AMPs experimentally, demonstrating broad-range antimicrobial activity.

Reduction in sequencing costs and the availability of high-throughput data facilitated by NGS have provided essential genetic resources to help expand fundamental knowledge of the biology and evolutionary history of an organism. Collectively, the present findings show that the combination of *in silico* and *in vitro* approaches could narrow down the identification of potential AMPs, and recent advances in both fields could be used to validate the applications of these 11 candidate AMPs as a template for further development as effective antibiotic therapeutics. Furthermore, these transcriptome sequencing results provide a genetic resource that should facilitate further comprehensive studies on the American cockroach.

## Supporting Information

S1 Fig Classification of Gene Ontology (GO) Subcategories Based on the WEGO Webserver at Level 3 for 2-Fold Up- and Down-Regulation. (TIF)

S2 Fig Classification of Gene Ontology (GO) Subcategories Based on the WEGO Webserver at Level 3 for Predicted Allergen Proteins Involved in Up- and Down-Regulation. (TIF)

S1 Table List of Novel Antimicrobial Peptides (AMPs). (XLSX)

S2 Table List of Putative Antimicrobial Peptides (AMPs). (XLSX)

S3 Table List of Allergens. (XLSX)
