# Supplementary figures and images for: De Novo Transcriptome Analysis and Detection of Antimicrobial Peptides of the American Cockroach Periplaneta americana (Linnaeus)
Source: PLoS One. 2016 May 11;11(5):e0155304. doi: 10.1371/journal.pone.0155304 (PMC4864078; doi:10.1371/journal.pone.0155304)

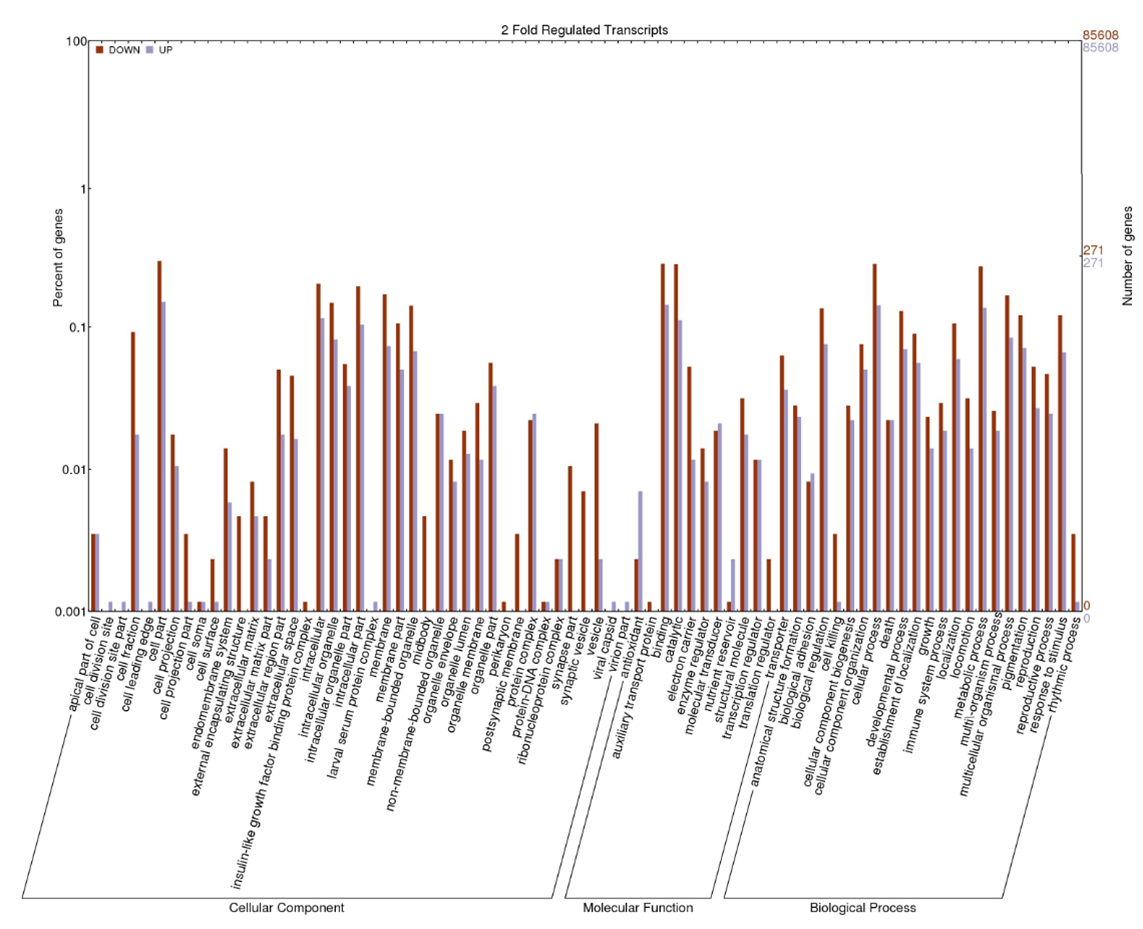

Supplement: S1 Fig — (TIF) [file pone.0155304.s001.tif]

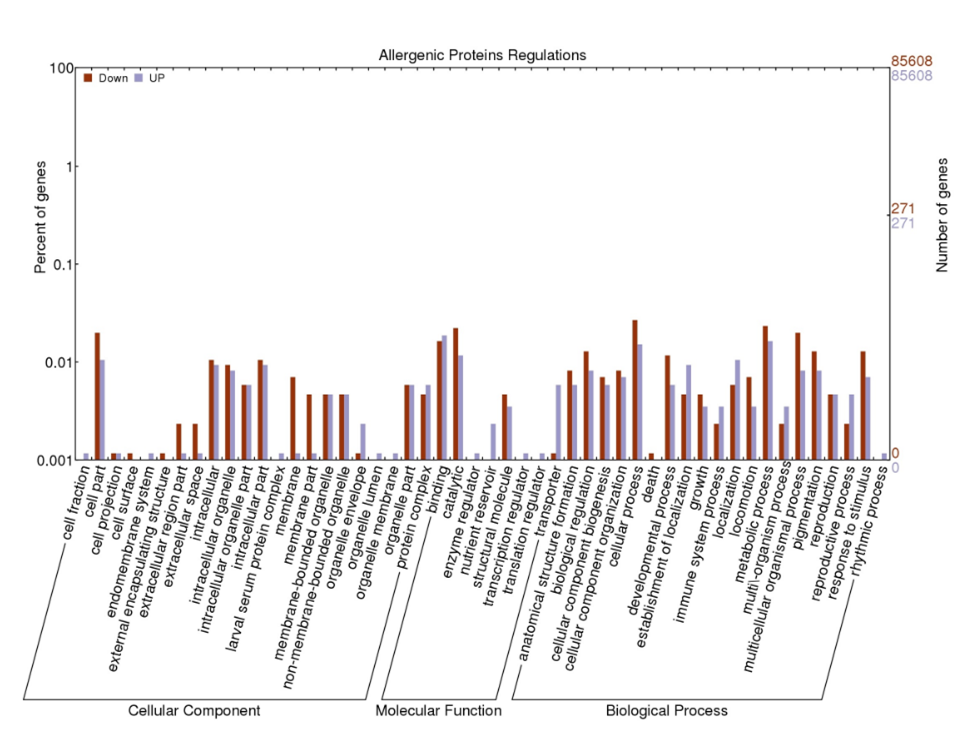

Supplement: S2 Fig — (TIF) [file pone.0155304.s002.tif]
